# Stretching the truth: length data highlight falsification of Japanese sperm whale catch statistics in the Southern Hemisphere

**DOI:** 10.1098/rsos.160506

**Published:** 2016-09-14

**Authors:** Phillip J. Clapham, Yulia V. Ivashchenko

**Affiliations:** Marine Mammal Laboratory, Alaska Fisheries Science Center, 7600 Sand Point Way NE, Seattle, WA 98115, USA

**Keywords:** whaling, illegal whaling, Southern Hemisphere, sperm whale, Japan, USSR

## Abstract

Falsification of reports on Japanese catches of sperm whales (*Physeter macrocephalus*) is known to have occurred at both land whaling stations and in North Pacific factory fleets. Here, we conduct an analysis of pelagic sperm whale catches in the Southern Hemisphere: we compare true Soviet length data from the *Yuri Dolgorukiy* factory fleet during 1960–1975 to data for the same period reported to the International Whaling Commission (IWC) by Japan. Prior to implementation of the International Observer Scheme (IOS) in 1972, the Soviet fleet killed 5536 females, of which only 153 (2.8%) were at or above the minimum legal length of 11.6 m. During the same period, Japan killed 5799 females and reported that 5686 (98.5%) were of legal size, with 88.5% of the entire length distribution reported as being between 11.6 and 12.0 m. This unrealistic distribution, together with the fact that Japanese fleets were supposedly able to catch 37 times the number of legal-sized females as the Soviet fleet, indicates extensive falsification of catch data by Japan. Further evidence of misreporting is that females >11.5 m dropped to 9.1% of the Japanese catch after 1971, when the IOS made cheating much more difficult. That 99.6% of 10 433 males in the pre-IOS catch were also reported to be of legal size, indicates that illegal catches were not confined to females. We caution that the Japanese sperm whale data in the IWC Catch Database are unreliable and should not be used in population assessments. The ease with which illegal catches were apparently made underscores the past failures of the IWC to effectively regulate whaling.

## 1. Introduction

The sperm whale (*Physeter macrocephalus*) is a globally distribution large cetacean which was the focus of intensive exploitation by whaling in both historical and modern times.

The systematic falsification by Japanese land whaling stations of catch numbers as well as biological data (length and sex) for sperm whales was previously demonstrated [1,2]. Recently, we used verified length data from Soviet whaling to demonstrate the unreliability of reported length data for catches of female sperm whales by Japanese factory fleets in the North Pacific, indicating extensive misreporting [3]. The Soviet data were contained in formerly secret whaling industry reports that were declassified following revelations regarding the extensive campaign of illegal whaling conducted by the USSR [4,5]. Data falsifications in both the land-based and pelagic Japanese fisheries in the North Pacific involved increasing the reported lengths of undersized animals to figures at or above the 11.6 m minimum length that was in force for sperm whales until 1972.

The International Whaling Commission (IWC) itself was at the time apparently aware that the minimum size limit was routinely being disregarded. For example, at its annual meeting in 1965, some Commissioners expressed great concern about the increased catches of sperm whales, but the Commission concluded as follows:

Moreover, while the minimum size limit—38 feet—should be enough to save the great majority of females, massive evidence was available to the Commission to show that this regulation was being broken on a large scale [6].

The report gives no indication of which countries were suspected of violating the rule, nor does it state what this ‘massive evidence’ was. It was also noted that the relatively low average production of oil per whale in catches by Japan and others indicated that many undersized animals were being caught [7]. However, no action was taken, either at that time or when new suspicions about pelagic catches of North Pacific sperm whales were raised 18 years later [8].

Here, we follow up on our North Pacific study [3] by conducting a similar comparative analysis of Japanese and Soviet sperm whale catches from the Southern Hemisphere (SH) and conclude that extensive falsification also occurred in Japanese factory fleets in that region.

## 2. Material and methods

Catch data reported to the IWC by the USSR for whaling operations were routinely falsified. However, true catch data were secretly recorded and retained for Soviet research purposes. Since their declassification, these have been accepted as the data of record by the IWC for both the North Pacific and the SH. As part of this, data preserved by the former Soviet whale biologist D. Tormosov are available as individual catch records (*n* = 51 476, all species) for the *Yuri Dolgorukiy* factory fleet, which operated in the SH between 1960 and 1975; this dataset contains records of date, location, sex and length for 23 938 sperm whales, of which 9689 were female. We obtained the data directly from Dr Tormosov, although they are also available in the IWC's Catch Database [9].

In this study, we conducted a similar comparative analysis to that reported for North Pacific sperm whales [3]. We used verified true data from the *Yuri Dolgorukiy* database to assess the reliability of the official Japanese catch statistics as compiled in the IWC's Catch Database (total Japanese records for SH sperm whales, 1960–1979 = 19 932) [8]. We focused on 1960–1975, the years for which both Soviet and Japanese length data are available; additional data are given for 1976–1979 for the Japanese fleets (*Yuri Dolgorukiy* ceased operations in 1975).

For the two data sources, we examined the number of female sperm whales whose length was equal to or greater than 11.6 m, the IWC's minimum allowable length for catches of this species (referred to here as legal-sized females (LSFs)).

In 1972, the IWC implemented the International Observer Scheme (IOS) [10]; at this time, independent inspectors began working on factory ships, thus (in theory) eliminating or greatly reducing illegal catches. Consequently, we hypothesized that if female length data were misreported by Japan prior to the IOS, the occurrence of female sperm whales measuring at or above the pre-1972 minimum legal length^1^ should be significantly lower when catches were independently inspected after the IOS was introduced.

We also examined length statistics for both nations' catches of male sperm whales (which in this species are much larger than females) before and after introduction of the IOS.

## 3. Results

### 3.1. Spatial distribution of whaling effort

In sperm whales, there is strong geographic segregation by sex and age [11]. Mature males frequent high latitudes, returning periodically to subtropical or tropical waters to mate. Females and immature whales of both sexes are found in much lower latitudes, typically those below 50 degrees. Because this segregation could affect the results of this analysis, we examined the spatial distribution of whaling effort for the two nations. It was very similar. Specifically, Japan and the USSR reported catching only 22 and 17 females, respectively, at high latitudes (those south of 50 S). All other female catches (Japan 7442; USSR 9672) were made at latitudes north of 50 S. Both nations took males in large numbers both north and south of 50 degrees.

### 3.2. Females

The number of female sperm whales killed by Japanese fleets and by the Soviet *Yuri Dolgorukiy* fleet, by year and length, are shown in table 1 (1960–1971) and table 2 (1972–1979, the post-IOS years). A more detailed breakdown of the length distribution of sperm whale catches by the two countries before and after the IOS is shown in table 3.

**Table 1.** Catches of female sperm whales in the Southern Hemisphere, by length, 1960–1971. Japanese data are from the IWC database [8]; Soviet data are from the factory fleet *Yuri Dolgorukiy* (source: D. Tormosov and IWC).

	Japan females			USSR females		
year	total	>11.5 m	%	total	>11.5 m	%
1960	6	6	100.0	57	1	1.8
1961	11	11	100.0	324	9	2.7
1962	362	361	99.7	588	14	2.4
1963	1850	1830	98.9	627	9	1.4
1964	2137	2114	98.9	790	19	2.4
1965	7	6	85.7	2093	33	1.6
1966	52	13	25.0	69	8	11.6
1967	0	0	—	353	5	1.4
1968	0	0	—	121	7	5.8
1969	137	132	96.4	19	4	21.1
1970	670	657	98.1	28	1	3.6
1971	567	556	98.1	467	43	9.2
total	5799	5686	98.1	5536	153	2.8

**Table 2.** Catches of female sperm whales in the Southern Hemisphere, by length, after implementation of the International Observer Scheme in 1972. Japanese data are from the IWC database [8]; Soviet data are from the factory fleet *Yuri Dolgorukiy* (source: D. Tormosov).

	Japan females			USSR females		
year	total	>11.5 m	%	total	>11.5 m	%
1972	398	81	20.4	1388	106	7.6
1973	240	18	7.5	518	14	2.7
1974	498	29	5.8	1263	43	3.4
1975	159	9	5.7	984	8	0.8
1976	115	0	0			
1977	168	11	6.5			
1978	0	0	0			
1979	87	4	4.6			
total	1665	152	9.1	4153	171	4.1

**Table 3.** Length distribution of female sperm whales taken by Japan and the USSR in the Southern Hemisphere, split by periods: 1960–1971 and following implementation of the IWC's International Observer Scheme in 1972. Lengths are given in metres.

nation	<11.1	11.1–11.5	11.6–12.0	12.1–12.5	12.6–13.0	13.1–13.5	>13.5	total
Japan 1960–1971	97	16	5133	473	50	26	4	5799
Japan 1972–1979	1125	388	131	20	1	0	0	1665
USSR 1960–1971	5090	293	129	18	4	1	1	5536
USSR 1972–1975	3321	661	155	15	1	0	0	4153

Before implementation of the IOS in 1972, the Soviet fleet killed 5536 females, of which only 153 (2.8%) were at or above the minimum legal length of 11.6 m. During the same period, Japan killed 5799 females but reported that 5686 (98.1%) were of legal size. This difference is highly significant (*χ*^2^ = 3716.3, d.f. = 1, *p* < 0.0001). Sorting lengths into half-metre bins (table 3) shows that 5133 of the Japanese whales—or 88.5% of the entire length distribution—were reported as being between 11.6 and 12.0 m.

Because introduction of the IOS in 1972 made illegal whaling much more difficult, we predicted that the frequency of LSFs in the Japanese catch data should markedly decrease in subsequent years and be more similar to the numbers taken by the USSR. This was the case: the proportion of LSFs declined from 98.1% (5686 of 5799) to 9.1% (152 of 1665) after 1971; compared with the equivalent pre-IOS Japanese numbers, the difference is highly significant (*χ*^2^ = 1078.3, d.f. = 1, *p* < 0.00001). The number of LSFs in the Soviet catch was small both before and after the IOS (2.8% and 4.1%, respectively).

**Figure 1.**
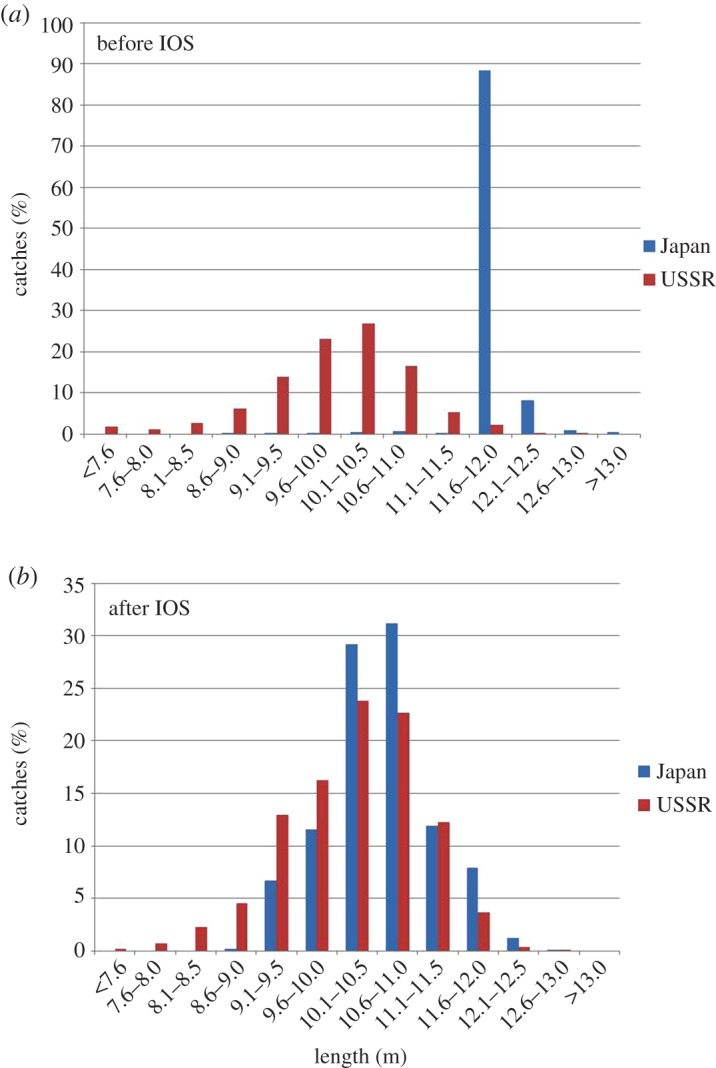
Length distributions (percentage of total catch) of female sperm whales killed in the Southern Hemisphere by Japanese fleets and the USSR's factory fleet *Yuri Dolgorukiy*: (*a*) 1960–1971 and (*b*) following implementation of the IWC's International Observer Scheme in 1972.

The difference between the two nations' post-IOS LSF figures was also highly significant (*χ*^2^ = 56.9, d.f. = 1, *p* < 0.0001). A comparison of both nations' length distributions before and after the IOS is shown in figure 1.

Similarly, very large females (arbitrarily defined here as those with a length greater than 12 m) were 22 times more commonly reported by Japan than were present in the Soviet catches (553 of 5799 versus 24 of 5536, respectively. This was not the case after 1972 (table 3).

### 3.3. Males

Of 10 479 males caught by Japan between 1960 and 1971, almost all (10 433 or 99.6%) were reported as legal size. The equivalent figures for the Soviet fleet for the same period were 8655 (total) and 6514 (75.3%, males above 11.5 m). The difference between the two nation's catches is significant (*χ*^2^ = 170.4, d.f. = 1, *p* < 0.00001).

After introduction of the IOS in 1972, the proportion of Japan's male catch consisting of whales above 11.5 m fell to 62.2% (1277 males from a total of 1989). The equivalent figure for the post-IOS Soviet catch of males was 72% (4027 from a total of 5590), a similar percentage to that nation's pre-IOS figure.

## 4. Discussion

As in the North Pacific [3], the present analysis highlights the implausibility of the length statistics for Japanese takes of sperm whales in the SH. Prior to implementation of the IOS, Japanese fleets were supposedly able to catch 37 times the number of LSFs as the Soviet fleet. The greatly skewed length distribution in the Japanese catch, together with the high frequency of large females, all indicate extensive falsification of length data. It is inconceivable that 88.5% of the entire length distribution of any species of whale would be contained within a single 50 cm bin; indeed, the expected normal distribution is evident in the true Soviet data, as well as in the Japanese data in the years following introduction of independent inspections (figure 1).

Nor can the difference between Soviet and Japanese length distributions be explained by geographical differences, as both whaling operations overlapped in their spatial and temporal hunting effort, and neither country caught more than a few females south of 50 S.

Unlike with the North Pacific analysis [3], we did not incorporate catcher boat effort into this study, but while that would undoubtedly result in minor changes to the figures reported here, the scale of the discrepancy between the Japanese and Soviet catch statistics is so large, and the Japanese length distribution so unrealistic, that it would not affect the conclusion.

Further evidence of illegal catches is that the difference between the pre- and post-IOS Japanese figures for the percentage of females larger than 11.5 m was significantly different, with far fewer in the catch after 1971. The overall distribution of length frequencies after 1971 was very similar between the two nations (figure 1); however, it is unclear why there were still significantly more LSFs in the post-IOS Japanese catch compared with that of the USSR.

Interestingly, there was a higher percentage of large females in the Soviet post-IOS catch compared with that before 1972, although the absolute number remained small. This is probably due to the presence of international observers preventing the take of numerous whales smaller than 9.2 m (the new minimum size limit introduced in 1972) as they had done previously, thus increasing the relative percentage of large females in the overall catch.

The phenomenon of ‘stretching’, whereby an undersized whale's reported length was increased, was well known throughout the industry. Stretching was employed to avoid an infraction penalty when an undersized animal's length was misjudged by a harpooner. However, as in the North Pacific, the SH data clearly reflect more than the occasional accidental take, and indicate that the catches made by Japan prior to the IOS involved the intentional taking of numerous under-sized whales.

That almost 100% of males killed were also reported to be above the minimum size limit indicates that it was not just females involved in the misreporting. Mature male sperm whales are much larger than females, but the family groups found in lower latitudes typically contain much smaller immature males. In all, 3660 of the males in the Japanese catch were killed between 30° and 40° S, and all but 27 of these were reported as above 11.5 m. It is not credible that catcher boat gunners were so unerringly accurate in their visual assessments of a whale's length to ensure that virtually every sperm whale caught (male and female) was of legal size.

Differing size selectivity between Japanese and Soviet whalers can be ruled out as an explanation for the large discrepancies in length statistics [3]. Soviet whalers were required to meet high production targets and thus actively sought the largest animals because of their high production value [5]. It was not that the Soviets ignored large females, but rather that they did not exist in the high numbers reported by Japan.

It is not known whether the Japanese misreporting involved falsifying catch numbers as well as length statistics, or changes in the sex of the catch (as routinely occurred in Soviet reports to IWC as well as in the Japanese coastal fishery [1,2,11]). Whatever the case, the Japanese data on length (and possibly also catch numbers) currently in the IWC Catch Database must be regarded as unreliable and should not be used in population assessments.

Given the widespread misreporting that is known to have occurred in the North Pacific, and the suspicions raised within the IWC at the time, the SH results are not surprising. Nonetheless, the misreporting by Japan, together with the USSR's 30-year campaign of illegal whaling, underscores the past failure of the IWC to manage whale stocks and effectively regulate whaling.

## Supplementary Material

Yuri Dolgorukiy sperm whale data.xlsx Soviet catch data used in this paper.

## Footnotes

1 The minimum length was reduced by IWC to 9.2 m in 1972; thus, smaller females were legal to catch after this date. However, because we wanted to investigate potentially illegal catches prior to implementation of the IOS, our analysis focused on the frequency of occurrence of 11.6+ m females before and after the introduction of inspection.

## Data accessibility

The Soviet dataset from this article has been uploaded as part of the electronic supplementary material. The Japanese data are available in the IWC Catch Database from the International Whaling Commission (contact Cherry Allison, cherry.allison@iwc.int).
